# Normative data for middle-aged Brazilians in Verbal Fluency (animals and FAS), Trail Making Test (TMT) and Clock Drawing Test (CDT)

**DOI:** 10.1590/1980-57642020dn14-010003

**Published:** 2020

**Authors:** Guilherme Almeida Carvalho, Paulo Caramelli

**Affiliations:** 1 Rede Sarah de Hospitais de Reabilitação, Belo Horizonte, MG, Brazil.; 2 Faculdade de Medicina da Universidade Federal de Minas Gerais, Belo Horizonte, MG, Brazil.

**Keywords:** neuropsychological tests, education, Brazil, reference standards, middle aged, testes neuropsicológicos, educação, Brasil, normas, meia-idade

## Abstract

Normative studies of neuropsychological tests were performed in Brazil in recent years. However, additional data are needed because of the heterogeneity of education of the Brazilian population.

**Objective:**

The present study provides normative data of executive function tests for middle-aged Brazilians and investigates the influence of age, sex, education and intelligence quotient (IQ) on performance in these tests.

**Methods:**

A total of 120 healthy staff and caregivers from a hospital were randomly selected and submitted to Fluency – animals and FAS, Trail Making Test (TMT) and Clock Drawing Test (CDT). They were divided into six groups of 20: two groups for age (45-54 and 55-64 years) and three groups for years of schooling (4-7; 8-11; 12+ years).

**Results:**

Normative data are presented in mean values and percentiles. Education influenced differences in the tests, except the CDT. Post hoc analyses revealed differences between the three educational levels on the TMT and FAS. Age differences emerged on the TMT and fluency letter F. Moderate correlation was found between schooling and results on TMT and Fluency. The correlations for IQ were similar.

**Conclusion:**

This study provides normative data for middle-aged Brazilians with four or more years of schooling in frequently used cognitive tests to assess executive functions. The results confirm the strong influence of education, even in the comparison between middle and higher levels.

## RESUMO

Estudos normativos de testes neuropsicológicos têm sido realizados no Brasil nos últimos anos. Entretanto, devido à heterogeneidade da educação da população brasileira, dados adicionais são necessários.

**Objetivo:**

O presente estudo fornece dados normativos de testes de funções executivas para brasileiros de meia-idade e investiga a influência da idade, sexo, educação e quociente de inteligência (QI) sobre o desempenho nestes testes.

**Métodos:**

Um total de 120 profissionais e cuidadores saudáveis de um hospital, selecionados aleatoriamente, foram submetidos aos testes de Fluência – animais e FAS – Teste de Trilhas (TT) e Teste do Desenho do Relógio (TDR). Foram divididos em seis grupos de 20, sendo dois por idade (45-54; 55-64) e três por anos de estudo (4-7; 8-11; 12+ anos).

**Resultados:**

Dados normativos são apresentados em médias e percentis. A educação influenciou as diferenças nos testes, exceto no TDR. Análises *post-hoc* evidenciaram diferenças entre os três níveis educacionais em TT e FAS. Surgiram diferenças por idade no TT e Fluência – letra F. Foi encontrada correlação moderada entre escolaridade e resultados do TT e Fluência. As correlações para QI foram semelhantes.

**Conclusão:**

O estudo fornece dados normativos para brasileiros de meia-idade, com quatro ou mais anos de escolaridade, em testes cognitivos frequentemente utilizados para avaliação de funções executivas. Os resultados confirmam a forte influência da educação, mesmo na comparação entre os níveis médio e superior.

Cognitive tests are essential tools in the neuropsychological assessment process. Although many normative studies were performed in Brazil in recent years, new efforts are necessary.^1^ Normative studies are generally expensive and require considerable logistical efforts.^2^ It is difficult to perform studies using large samples. Therefore, normative studies are important, even if these studies are not original for that population. This need is greater when we consider the size and educational heterogeneity of the Brazilian population. In the 2015 Brazilian demographic census, 42.3% of people aged over 25 years had eight or less years of schooling.^3^ Most normative studies worldwide, and some in Brazil, are performed using participants with 11 or more years of schooling.

Neuropsychological assessment is a comprehensive process involving more than the interpretation of patients’ tests scores compared to the norms for the population. However, normative data make great differences in some cases, and in these cases, there is some risk in comparing a patient with a low educational level with others with higher levels of schooling. For example, it is possible to achieve false positive results in the investigation of cognitive impairment or dementia. The implications of this type of mistake may have serious consequences.^4^^,^ 5

Mitrushina et al.^2^ performed a comprehensive review and showed that age and educational level were the main factors in the variability of test results in healthy populations. Although this provided strong evidence, these authors highlight the importance of specific data for each population. They also discuss the importance of analyzing the factors related to current cognitive ability as an influence on neuropsychological test results. In this sense, the intelligence quotient (IQ) may provide this type of information.

Most of the studies were conducted with elderly populations because of the greater incidence of dementia in this stage of life. However, there are many neurological or psychiatric disorders that emerge in middle-aged populations,^6^ and cognitive changes across the life cycle are a slow process that may begin in the second and third decades.^7^ Therefore, it is important to have references of the cognitive profiles of middle-aged adults to identify signs of pathological cognitive loss in this age group.

In order to contribute to clinical practice, commonly used cognitive tests were chosen in the present study. These tests are often used because of their utility to assess executive functions in clinical settings. Besides, several studies aiming to evaluate the cognitive profile of adults and elderly people include some of these tests in their battery.^8^^-^ 11

Fluency tests are widely used because of their sensitivity for executive functions,^12^ detection of dementia,^13^ and fast application. Among the studies in Brazil to obtain normative data for the “animal” class, some studies were directed to the elderly population.^11^^,^ 13 ^,^ 14 Another study included a very large adult age range of 15 and 64 years.^15^ The cutoffs ranged from 9 to 13 primarily depending on education level.

One study of phonemic fluency (FAS) in Brazil was designed to verify the influence of education and age on test results and included an elderly sample.^16^ Education was the principal influence in these results. Another study investigated the age effect^17^ in a sample with 10 years or more of schooling.

The Trail Making Test (TMT) is one of the most widely used tests worldwide,^18^ and it demonstrates accuracy in the detection of signs of neuropsychological sequelae of brain lesions.^19^ The cognitive abilities required to perform the test are visual search, motor/perceptual speed, speed of processing and general intelligence. Executive functions are more demanding in part B of the test.^20^ There are few normative studies for neuropsychological tests in general. However, TMT has a larger number of studies because of its popularity.^2^

In Brazil, Campanholo et al.^21^ performed a normative study using a large and representative sample from different Brazilian regions. However, Mitrushina et al.^2^ demonstrated great variability in scores between normative studies for this test. Therefore, it is important to have references for different population profiles. Other studies^22^^,^ 23 confirmed the strong influence of age and education on results, also contributing to normative data.

The Clock Drawing Test (CDT) is an important non-linguistic tool for dementia screening.^7^ Pinto and Peters^24^ performed an extensive review and concluded that the CDT was more reliable for the evaluation of moderate or severe dementia. Leung et al.^25^ showed that the test fits better with intermediate levels of education. One Brazilian study in an elderly population also suggested caution in the use of this test for the screening of dementia in people with less than four years of schooling.^26^

The short review above demonstrates that there are more references in Brazil for these tests published in the last years. However, new studies are relevant because of the strong influence of cultural factors on cognition. Therefore, the present study aims to provide normative data for middle-aged Brazilians in Fluency tests (animals and FAS), Trail Making Test and Clock Drawing Test and to investigate the influence of age, sex, education and IQ on the results.

## METHODS

### Study design: observational, cross-sectional

- ***Setting:*** The Hospital Sarah and Universidade Federal de Minas Gerais – UFMG ethics committees approved the study. Data collection was conducted at the Hospital Sarah in Belo Horizonte (MG), Brazil between May 2015 and October 2016. The first author performed all of the evaluations. The tests were administered in an appropriate and quiet room that lacked external stimuli. The researcher had extensive experience in the application of cognitive tests.
- ***Participants:*** Middle-aged Brazilians, between 45 and 64 years, with at least four years of schooling, were invited to participate in the study. Individuals with neurological and psychiatric disorders, whose symptoms could cause cognitive impairment at the time of the tests, and subjects with hearing or visual impairment, were excluded. We also excluded individuals who reported the use of psychoactive drugs within three weeks prior to the administration of the tests. Subjects who reported alcohol dependence or were using illicit drugs were also excluded.

The Mini-Mental Sate Examination (MMSE)^27^ was used as the study entry criteria. Individuals below the established education-adjusted cutoff points were excluded (24 for 4 to 7 years of schooling and 26 for 8 or more years).^28^^,^ 29

To preserve anonymity, participants were given an identifier (e.g., P1, P2). Only the author responsible for the procedures had access to the names.

- ***Variables:*** Dependent variables were the neuropsychological tests scores, and independent variables were years of schooling, age, sex and IQ.
- ***Sample:*** The sample was selected from the two populations of the hospital cited above: hospital staff and inpatients’ relatives and caregivers. The final sample was comprised of 120 adults divided into two age groups (45-54 and 55-64 years). Each age group was divided into three groups by schooling (4-7, 8-11 and 12+ years of schooling). The groups were divided equally using the two criteria into six groups with 20 participants each. The staff population was invited from a random list. The relatives and caregivers were selected weekly from the inpatient list. We established that even numbers on the list would be called initially in order to preserve randomness.

### Procedures

- ***Invitation:*** The objective of the study was explained in general terms at this time. Interviews for subjects who agreed were scheduled generally within one week.
- ***Interview:*** After signing the consent form, a semi-structured interview was performed. A history of possible neurological and psychiatric illnesses, health conditions, medications in use, possible history of alcohol and drug use, auditory and visual acuity, school history and income were collected. For schooling, the Brazilian law that was valid until 2006 was taken into consideration, which was when most of the participants attended school. We considered four years of schooling for participants who declared to have completed the primary course (near elementary school in the United States), eight years for subjects who completed the first grade (like junior high school) and 11 years for the second grade (like high school). Twelve years of study were considered for subjects who completed one year of college.

MMSE^27^ and Mini-International Neuropsychiatry Interview (MINI)^30^^,^ 31 were performed after the interview. MINI is a structured interview designed to provide a better characterization of psychiatry disorders according to DSM-IV criteria. Diagnostic modules A (Major Depressive Disorder), J (Alcohol dependence) and O (Generalized anxiety Disorder) were used to exclude the most frequent psychiatric disorders.

### Wechsler Abbreviated Scale of Intelligence (WASI) and neuropsychological tests application

For the participants who satisfied the selection criteria, another session was scheduled for the application of the WASI and neuropsychological tests. WASI was used as an intelligence measure, which was one of the independent variables proposed in the study.^32^ The version with two subtests (Vocabulary and Matrix Reasoning) was chosen.

### Neuropsychological tests

- ***Trail Making Test:*** This task consists of connecting circles numbered 1 to 25 in part A (TMT-A) and numbers and letters in part B (TMT-B) in alternating sequences (e.g., 1-A-2-B-3...). The instructions from Strauss et al.^7^ were used, and the spatial distribution was like Santos.^33^ The letter K was not present in the alphabet in Brazil when most of the participants were in school. Therefore, a form without K was used, but the 25 circles were preserved up to the letter M. Information on the lack of the letter K was added on instructions.

In general, we allowed 180 seconds to complete part A and 300 seconds to complete part B. The criteria to interrupt the task were exceeding the maximum time or making more than five mistakes. We registered 301 seconds for subjects who exceeded the time or did not complete the task in part B. The time in seconds and ratio of part B to part A were used as scores (TMT-B/A).

- ***Fluency tests:*** The animal category was used for the semantic test (FL ANIM), and the letters F (FL F), A (FL A) and S (FL S) were used for the phonemic test. The instructions for semantic fluency were described in Brucki et al.^15^ The participants said as many animal as possible in one minute. Any kind of real animal was accepted. For phonemic fluency, the reference was the instructions suggested by Machado et al.,^16^ with one minute for each letter. Proper nouns, such as names of peoples, cities or countries, were not accepted, neither was the same word with a different suffix. The score was the number of words generated in a given time and the sum of the three letters (FL FAS).
- ***Clock drawing test (CDT):*** The instructions for the free-drawing version were described in Strauss, Sherman and Spreen (2006).^7^ A sheet of A4 paper was displayed in an upright position, and the participants were asked to draw the face of a watch in a large size and place all of the numbers. They were then prompted to set the time to 10 after 11. The scoring system proposed by Sunderland was used for interpretation, and the scores ranged from 0 to 10.^7^

### Statistical analysis

The Shapiro-Wilk test was used to verify the hypothesis of frequency distribution normality.

Regarding education, in which three groups were compared, the ANOVA test was chosen for normal distribution and Kruskal-Wallis (K-W) for non-normal distribution values. Post hoc analyses were performed using the Least Significance Difference (LSD) test. The Mann-Whitney (M-W) test was used for the Kruskal-Wallis test, with Bonferroni correction for p-values. In paired comparisons, which were used to analyze sex and age, Student’s t-test or the Mann-Whitney test were used, according to the frequency distribution.

Age, years of schooling and IQ were also analyzed as continuous variables. Spearman’s rank correlation coefficient (r_s_) between test results and the above variables was used. To interpret the correlations, the classification suggested by Siqueira and Tibúrcio^34^ was followed: 0 to 0.4, weak; 0.4 to 0.7, moderate; and 0.7 to 1.0, strong. The same graduation applied to negative values.

All analyses were performed using the statistical software SPSS, version 20.0. The level of significance considered was p<0.05. In the cases in which Bonferroni’s correction was performed, p<0.017 was considered significant.

## RESULTS

### Participants

Overall, 153 individuals were invited to participate. There were 13 non-respondents and 18 exclusions: 12 due to the use of medications with potential negative cognitive effects; one for neurological disease; one was outside the age group; and four failed to meet the diagnostic criteria of the MINI interview. From the 122 participants included, two gave up during the third stage, which was related to the application of neuropsychological tests. Table 1 shows the general characterization of the sample.

**Table 1:** Description of participants.

Groups (years of schooling / age)	Age^1^	Years of schooling^1^	Sex^2^	QI^1^	n
4-7 / 45-64	49.85 ± 3.08	5.15 ± 1.09	10 (50%)	81.4 ± 14.15	20 (16.7%)
4-7 / 55-64	59.25 ± 2.92	4.95 ± 1.36	13 (65%)	77.2 ± 9.53	20 (16.7%)
8-11 / 45-64	49.65 ± 2.46	10.70 ± 0.80	11 (55%)	96.45 ± 11.18	20 (16.7%)
8-11 / 55-64	57.90 ± 1.80	10.10 ± 1.33	15 (75%)	91.90 ± 16.46	20 (16.7%)
≥ 12 / 45-64	48.80 ± 2.80	15.55 ± 2.39	15 (75%)	110.85 ± 11.13	20 (16.7%)
≥ 12 / 55-64	58.45 ± 2.28	16.85 ± 2.66	15 (75%)	111.25 ± 10.50	20 (16.7%)

Source: authors.

1 Mean values (MV) ± standard deviation (SD).

2 Percentage of females.

### Normative data

The results in neuropsychological tests, except the CDT, are shown in Tables 2 and 3, in means, standard deviations and percentiles. Part of the results did not have a normal distribution, and the use of percentile (P) was recommended, with P10 being indicative of impairment.^2^

**Table 2:** Normative data for Trail Making Test.

		45-54 years old				55-64 years old		
		4-7*	8-11	12+		4-7	8-11	12+
TMT - A	MV ± SD	52.85 ± 19.99	39.05 ± 12.86	34.05 ± 9.94		65.75 ± 22.21	44.6 ± 10.68	35.95 ± 10.07
	P95	22	20	16		34	24	19
	P90	30	25	22		35	31	20
	P75	37	27	24		49	38	29
	Median	48	40	35		64	44	34
	P25	66	45	43		73	52	45
	P10	85	51	45		107	56	50
	P05	91	77	54		119	72	54
TMT - B	MV ± SD	175.8 ± 77.84	99.25 ± 53.82	78.3 ± 32.87		208.4 ± 62.74	126.65 ± 58.45	86.65 ± 23.48
	P95	80	51	37		99	53	47
	P90	88	52	39		120	73	47
	P75	101	70	49		175	91	72
	Median	158	88	77		195	106	92
	P25	247	113	95		257	155	108
	P10	301	145	141		301	206	112
	P05	301	293	155		301	296	122
TMT - B/A	MV ± SD	3.44 ± 1.26	2.55 ± 0.84	2.33 ± 0.80		3.35 ± 1.19	2.82 ± 0.94	2.52 ± 0.81
	P95	1.4	1.4	1.4		1.8	1.5	1.3
	P90	2.2	1.7	1.5		2.2	1.7	1.6
	P75	2.7	2.0	1.6		2.7	2.1	2.0
	Median	3.2	2.3	2.1		3.2	2.7	2.4
	P25	4.0	3.2	2.6		4.0	3.4	3.0
	P10	5.6	3.9	4.0		4.3	4.1	3.4
	P05	6.7	4.4	4.1		7.3	5.0	4.8

Source: authors. n = 20 for each group.

* 4-7. 8-11 and 12+ refer to years of schooling.

**Table 3:** Normative data for fluency tests (animals and FAS).

		45-54 years old				55-64 years old		
		4-7	8-11	12+		4-7	8-11	12+
Fluency animals	MV ± SD	15.6 ± 4.63	18.6 ± 3.62	22.1 ± 5.11		15.4 ± 3.68	15.85 ± 4.80	20.05 ± 5.13
	P95	24	26	33		22	23	28
	P90	24	24	30		21	23	27
	P75	18	21	27		18	21	24
	Median	16	18	21		16	17	20
	P25	12	16	17		13	11	16
	P10	11	13	16		9	9	12
	P05	7	11	16		8	9	11
Fluency letter F	MV ± SD	10.85 ± 4.57	12.7 ± 2.77	14.4 ± 3.42		9.75 ± 3.48	10.2 ± 3.79	13.4 ± 5.18
	P95	21	18	20		16	18	24
	P90	17	17	20		14	17	19
	P75	15	16	17		12	14	18
	Median	11	12	15		10	9	12
	P25	7	11	11		8	8	10
	P10	5	9	10		3	5	5
	P05	5	9	9		2	4	3
Fluency letter A	MV ± SD	9.55 ± 4.65	12.3 ± 3.25	12.9 ± 3.89		8.65 ± 4.07	10.05 ± 3.14	12.2 ± 3.59
	P95	21	18	20		16	18	24
	P90	16	17	18		16	15	17
	P75	14	15	17		11	12	15
	Median	8	12	12		8	10	13
	P25	6	10	10		5	8	9
	P10	4	8	7		4	6	7
	P05	3	7	5		3	5	6
Fluency letter S	MV ± SD	9.4 ± 4.5	11.55 ± 3.24	13.1 ± 3.89		8.6 ± 3.99	10.1 ± 2.57	13.45 ± 4.24
	P95	19	20	20		16	16	22
	P90	16	16	20		15	14	20
	P75	13	14	16		11	12	16
	Median	9	11	13		9	10	13
	P25	7	10	10		6	8	10
	P10	2	7	8		3	7	8
	P05	2	6	6		0	7	6
Fluency FAS	Média ± DP	29.8 ± 12.73	36.55 ± 7.19	40.4 ± 8.61		27 ± 9.47	30.35 ± 7.75	39.05 ± 11.55
	P95	60	51	55		45	48	61
	P90	48	48	50		41	44	53
	P75	38	43	48		34	36	48
	Median	29	36	42		24	30	42
	P25	21	30	31		21	24	30
	P10	14	28	29		12	21	20
	P05	10	28	25		9	20	18

Source: authors. n = 20 for each group.

In the case of the CDT, eight of the 120 participants obtained a score of 7, and one obtained a score of 6. These scores were similarly distributed among the school levels: three at the basic level; four at the intermediate level; and two at the higher level. From the total number of participants, 82% scored 10.

### Education

The result analyses between groups showed significant differences for almost all tests, except the CDT, as shown in Table 4.

**Table 4:** Comparisons of groups by educational level.

	4-7 MV ± SD Median (Q*1; Q3)	8-11 MV ± SD Median (Q1; Q3)	12+ MV ± SD Median (Q1; Q3)	p
TMT- A	59.3 ± 21.86 63 (73; 41.25)	41.83 ± 12 41.5 (47.75; 33.25)	35 ± 9.92 34 (42.75; 28.25)	<0.001^1^
TMT- B	192.10 ± 71.71 191.5 (252.75; 129.5)	112.95 ± 57.17 95.5 (124.25; 80.75)	82.48 ± 28.51 80.5 (101.5; 56.25)	<0.001^1^
TMT- B/A	3.39 ± 1.21 3.21 (3.96; 2.68)	2.69 ± 0.89 2.5 (3.29; 2.03)	2.43 ± 0.8 2.31 (2.78; 1.85)	<0.001^1^
FL ANIM	15.5 ± 4.13 16 (12.25; 18)	17.23 ± 4.42 18 (14; 20.75)	21.08 ± 5.16 20 (17; 25)	<0.001^2^
FL F	10.3 ± 4.05 10 (8; 13)	11.45 ± 3.52 11 (9; 14)	13.90 ± 4.36 13.5 (11; 17.75)	<0.001^2^
FL A	9.10 ± 4.34 8 (6; 11)	11.18 ± 3.55 11 (8; 14)	12.55 ± 3.71 12 (9.25; 15)	<0.001^1^
FL S	9.00 ± 4.22 9 (6; 12)	10.83 ± 2.98 10 (8.25; 12.75)	13.28 ± 4.02 13 (10; 16)	<0.001^1^
FL FAS	28.40 ± 11.16 27 (21; 35.75)	33.45 ± 8.02 31 (28; 40.75)	39.73 ± 10.68 41.5 (30.25; 48.25)	<0.001^2^
TDR	9.38 ± 1.10 10 (8.25; 10)	9.58 ± 0.98 10 (10; 10)	9.75 ± 0.78 10 (10; 10)	0.157^1^

Source: authors. n = 40 for each group.

1 Kruskall Wallis;

2 ANOVA.

* Q: Quartile.

Post hoc analyses revealed a difference in comparisons between the three school levels in parts A and B. In the derived score (B/A), the comparison between the school levels 8-11 and 12+ was not significant. In the category fluency test (animals), the comparison between 4-7 and 8-11 was not significant. In the sum of FAS, the difference was significant in all pairs. In the analyses of each letter, there were no differences between 4-7 and 8-11 for the letters F and S, and between 8-11 and 12+ for letter A.

### Age

Comparisons between the two age groups demonstrated influence on parts A (p = 0.036) and B (p = 0.026) of TMT (M-W) and the letter F of FAS (p = 0.047), with better performances in the younger age group (t test).

### Sex

A difference was found in the letter F of FAS (t test), with better performance for men (p = 0.014).

### Correlations

Table 5 shows a comparison of the main results. Similar moderate correlations were observed for the variables years of study and IQ, in most of the results, except for CDT. The correlations were low and negative for age. A high correlation (r_s_ = 0.775) was found between the independent IQ variable and years of schooling.

**Table 5:** Spearman's correlations (rs) between tests results and independent variables.

Tests	Years of schooling	IQ	Age**	Age's p
TMT - A	-0.570*	-0.667*	**0.228**	**0.012**
TMT - B	-0.655*	-0.772*	**0.213**	**0.019**
TMT B/A	-0.351*	-0.415*	0.038	0.678
FL ANIM	0.463*	0.484*	**-0.183**	**0.046**
FL F	0.385*	0.464*	**-0.198**	**0.030**
FL A	0.388*	0.480*	-0.137	0.137
FL S	0.448*	0.503*	-0.042	0.645
FL FAS	0.473*	0.561*	-0.142	0.122
CDT	0.155†	0.281§	-0.080	0.384

Source: Authors. n = 120.

* p<0,001.

† p = 0,191;

§ p = 0,002.

** In age, there were highlighted the significative correlations.

## DISCUSSION

The present study provides normative data for tests that are frequently used in Clinical Neuropsychology, with references for the population with four or more years of study, which is essential for countries such as Brazil because of the educational heterogeneity of the population. The quality of the data obtained was related to some aspects, such as the established exclusion criteria, the random selection of participants, the rigor in the application of the tests and the inclusion of IQ as an independent variable.

The option for middle-aged adults offers normative data for an age group that is generally less investigated, once references for the elderly are more common. However, the investigation of the influence of age became restricted because the composition of the groups allowed better analysis of education. These restrictions are common, except in a few studies with larger samples. Comparisons with other normative studies are not easy because of differences in the education and age ranges, and the way the data are shown (e.g., mean values or percentiles).

A test designed as a short scale (WASI) was used to obtain IQ. Some studies used an estimate based on two subtests of the Wechsler Adult Intelligence Scale (WAIS). This strategy is often used, but it requires greater care in interpretation.^35^ The short scale also does not replace the full scale, but it offers norms from a sample that was evaluated using that form of the test. The IQ as an independent variable was included in the present study following Mitrushina et al. recommendation.^2^ However, we didn’t find substantial differences between the influences of IQ and education. Considering the cost of including IQ evaluation, the researcher should think about its necessity.

This study has limitations because it was restricted to staff and patient caregivers from a hospital unit. Considering the dimensions of our country and its cultural variety, multicenter studies may provide additional contributions on the influence of other sociodemographic aspects beyond age and education.

### Trail making test

Our results indicated the influence of education in parts A and B. Unlike Campanholo et al.,^21^ there was also a difference in results between the intermediate and higher educational levels in both parts. For 0-4 years of schooling on TMT B, the subjects of the study of Campanholo et al. had a mean value of 88.67 for individuals aged 50-59 years (n = 9). For subjects aged 60-69 years, the mean value was 173.03 (n = 31). In the range of 4-7 years of schooling, our median was 158 for participants aged 45-54 years and 195 for those with ages 55-64. Therefore, our data are consistent with the results of the 60-69 age range. In that previous study, an IQ of 80 or less was an exclusion criterion, and a sample with better cognitive potential was selected. This analysis is an example of how differences in studies influence tests results.

Fernandez and Marcopoulos made a comparison among normative studies in several countries. The study used only age as parameter, limiting the results. However, even when comparing countries with similar school levels, such as the United States and Sweden, there are differences in the times considered normal or pathological.^36^ Considering our experience in the study, the application of part B should be avoided for populations with less than eight years of schooling. Some participants in this subsample did not complete the test. Many of the participants who completed the test were very slow and expended considerable effort.

### Fluency tests

This study indicated a greater influence of education in the category (animals) and phonemic (FAS) tests. The age analysis only showed differences in the letter F of FAS. These data cannot be compared with those from other studies which evaluated the influence of age and education, such as in Tombaugh et al.,^37^ due to the focus on a middle-aged population.

In the Brazilian studies for Fluency animals that provide data for basic and middle educational levels,^13^^,^ 15 cutoffs are near of our 10^th^ percentile: 9 to 13 animals. In Mitrushina meta-analysis for the FAS test, the predictive scores for individuals aged 45 to 64 years (average schooling of 14,31 ± 2,33 years), were between 41 and 45.^2^ These results are near to ours. We found median values of 42 for 12 or more years of schooling.

Concerning the applicability of FAS for the group with less than eight years of study, the 10^th^ percentile for this group ranged from 2 to 5 words per minute. In clinical practice, these results may hinder the interpretation of scores. This discrimination is more viable in the category fluency test.

### Clock-Drawing Test

The CDT was the only one that did not show an influence of education on the results, which reinforces evidence of its applicability in populations with more than four years of schooling. However, evaluation methods should be considered. The Sunderland scale,^7^ used in this study, may be less discriminatory for some drawing details because it focuses on patients with more severe spatial problems. In a study using qualitative analysis^38^, differences between participants with more and less than eight years of study were verified.

In conclusion, we hope that the present study encourages new normative studies in Brazil to further improve the quality and confidence of neuropsychological assessment in our country.

## References

[1] Malloy-Diniz LF, Fuentes D, Mattos P, Abreu N (2010). Avaliação neuropsicológica.

[2] Mitrushina M, Boone KB, Razani J, D'Elia LF (2005). Handbook of Normative Data for Neuropsychological Assessment.

[3] IBGE, Diretoria de Pesquisas, Coordenação de Trabalho e Rendimento Pesquisa Nacional de Amostra de Domicílios 2007/2015 [Internet].

[4] Parente MA, Scherer LC, Zimmermann N, Fonseca RP (2009). Evidências do papel da escolaridade na organização
cerebral. Rev Neuropsicol Latinoam.

[5] Rosselli M, Ardila A (2003). The impact of culture and education on non-verbal
neuropsychological measurements: A critical review. Brain Cogn.

[6] Schlindwein-Zanini R, Malloy-Diniz LF, Fuentes D, Mattos P, Abreu N (2010). Avaliação neuropsicológica de adultos. Avaliação Neuropsicológica.

[7] Strauss EH, Sherman EMS, Spreen O (2006). A Compendium of Neuropsychological Tests - Administration, Norms And
Commentary.

[8] Passos VM de A, Gatti L, Bensenor I, Tiemeier H, Ikram MA, Figueiredo RC (2015). Education plays a greater role than age in cognitive test
performance among participants of the Brazilian Longitudinal Study of Adult
Health (ELSA-Brasil). BMC Neurol.

[9] Farina M, Breno Costa D, Webber de Oliveira JA, Polidoro Lima M, Machado WDL, Moret-Tatay C (2019). Cognitive function of Brazilian elderly persons: longitudinal
study with non-clinical community sample. Aging Ment Heal.

[10] César KG (2014). Estudo da prevalência de comprometimento cognitivo leve e demência na
cidade de Tremembé, estado de São Paulo.

[11] Silva TBL, Yassuda MS, Guimarães VV, Florindo AA (2011). Fluência verbal e variáveis sociodemográficas no processo de
envelhecimento: Um estudo epidemiológico. Psicol Reflexão e Crítica.

[12] Ross TP, Calhoun E, Cox T, Wenner C, Kono W, Pleasant M (2007). The reliability and validity of qualitative scores for the
Controlled Oral Word Association Test. Arch Clin Neuropsychol.

[13] Caramelli P, Carthery-Goulart MT, Porto CS, Charchat-Fichman H, Nitrini R (2007). Category Fluency as a Screening Test for Alzheimer Disease in
Illiterate and Literate Patients. Alzheimer Dis Assoc Disord.

[14] Bertolucci PHF, Okamoto IH, Brucki SMD, Siviero MO, Toniolo J, Ramos LR (2001). Applicability of the CERAD neuropsychological battery to
brazilian elderly. Arq Neuropsiquiatr.

[15] Brucki SMD, Malheiros SMF, Okamoto IH, Bertoluccl PHF (1997). Dados normativos para o teste de fluência verbal categoria
animais em nosso meio. Arq Neuropsiquiatr.

[16] Machado TH, Fichman HC, Santos EL, Carvalho VA, Fialho PP, Koenig AM (2009). Normative data for healthy elderly on the phonemic verbal fluency
task - FAS. Dement Neuropsychol.

[17] Steiner VAG, Mansur LL, Brucki SMD, Nitrini R (2008). Phonemic verbal fluency and age - A preliminary
study. Dement Neuropsychol.

[18] Rabin LA, Barr WB, Burton LA (2005). Assessment practices of clinical neuropsychologists in the United
States and Canada: A survey of INS, NAN, and APA Division 40
members. Arch Clin Neuropsychol.

[19] Lezak MD, Howieson DB, Bigler ED, Tranel D (2012). Neuropsychological assessment.

[20] Sánchez-Cubilllo I, Periáñez JA, Adrover-Roig D, Rodríguez-Sánchez JM, Ríos-Lago M, Tirapu J (2009). Construct validity of the Trail Making Test: role of
task-switching, working memory, inhibition/interference control, and
visuomotor abilities. J Int Neuropsychol Soc.

[21] Campanholo KR, Romão MA, Machado MAR, Serrao VT, Coutinho DGC, Benute RGG (2014). Performance of an adult Brazilian sample on the Trail Making Test
and Stroop Test. Dement Neuropsychol.

[22] Hamdan AC, Hamdan EMLR (2009). Effects of age and education level on the Trail Making Test in a
healthy brazilian sample. Psychol Neurosci.

[23] Zimmermann N, Cardoso CO, Kristensen CH, Fonseca RP (2017). Brazilian norms and effects of age and education on the Hayling
and Trail Making tests. Trends Psychiatry Psychother.

[24] Pinto E, Peters R (2009). Literature review of the Clock Drawing Test as a tool for
cognitive screening. Dement Geriatr Cogn Disord.

[25] Leung JCW, Lui VWC, Lam LCW (2005). Screening for early Alzheimer' s disease in elderly chinese
patients using the Chinese Clock Drawing Test. Hong Kong J Psychiatry.

[26] Lourenço RA, Ribeiro-Filho ST, Moreira IFH, Paradela EMP, De Miranda AS (2008). The Clock Drawing Test : performance among elderly with low
educational level. Rev Bras Psiquiatr.

[27] Folstein MF, Folstein SE MP (1975). Mini-mental state. A grading the cognitive state of patiens for
the clinician. J Psychiatr Res.

[28] Almeida OP (1998). Mini Exame do Estado Mental e o diagnóstico de demência no
Brasil. Arq Neuropsiquiatr.

[29] Bertolucci PHF, Brucki SMD, Campacci SR, Juliano Y (1994). O Mini-Exame do Estado Mental em uma população geral: impacto da
escolaridade. Arq Neuropsiquiatr.

[30] Amorim P (2000). Mini International Neuropsychiatric Interview ( MINI ): validação
de entrevista breve para diagnóstico de transtornos mentais. Rev Bras Psiquiatr.

[31] Lecrubier Y, Weiller E, Hergueta T, Amorim P, Bonora LI, Lépine JP (2002). M.I.N.I: Mini International Neuropsychiatric Interview - Brazilian.
Version 5.0.0.

[32] Wechsler D, Trentini CM, Yates DB, Heck VS (2014). Escala Wechsler Abreviada de Inteligência - WASI : Manual.

[33] Santos J (2011). Validação do teste de trilhas - B (trail making test - B) para uso em
pacientes brasileiros com câncer em cuidados paliativos.

[34] Siqueira AL, Tibúrcio JD (2011). Estatística na área da saúde: conceitos, metodologia, aplicações e
prática computacional.

[35] Wagner F, Pawlowski J, Yates DB, Camey SA, Trentini CM (2010). Viabilidade da estimativa de QI a partir dos subtestes
Vocabulário e Cubos da WAIS-III. Psico-USF.

[36] Fernández AL, Marcopulos BA (2008). A comparison of normative data for the Trail Making Test from
several countries: Equivalence of norms and considerations for
interpretation. Scand J Psychol.

[37] Tombaugh TN, Kozak J, Rees L (1999). Normative data stratified by age and education for two measures
of verbal fluency: FAS and animal naming. Arch Clin Neuropsychol.

[38] Fabricio Teixeira A, Aprahamian I, Yassuda Sanches M (2014). Qualitative analysis of the Clock Drawing Test by educational
level and cognitive profile. Arq Neuropsiquiatr.

